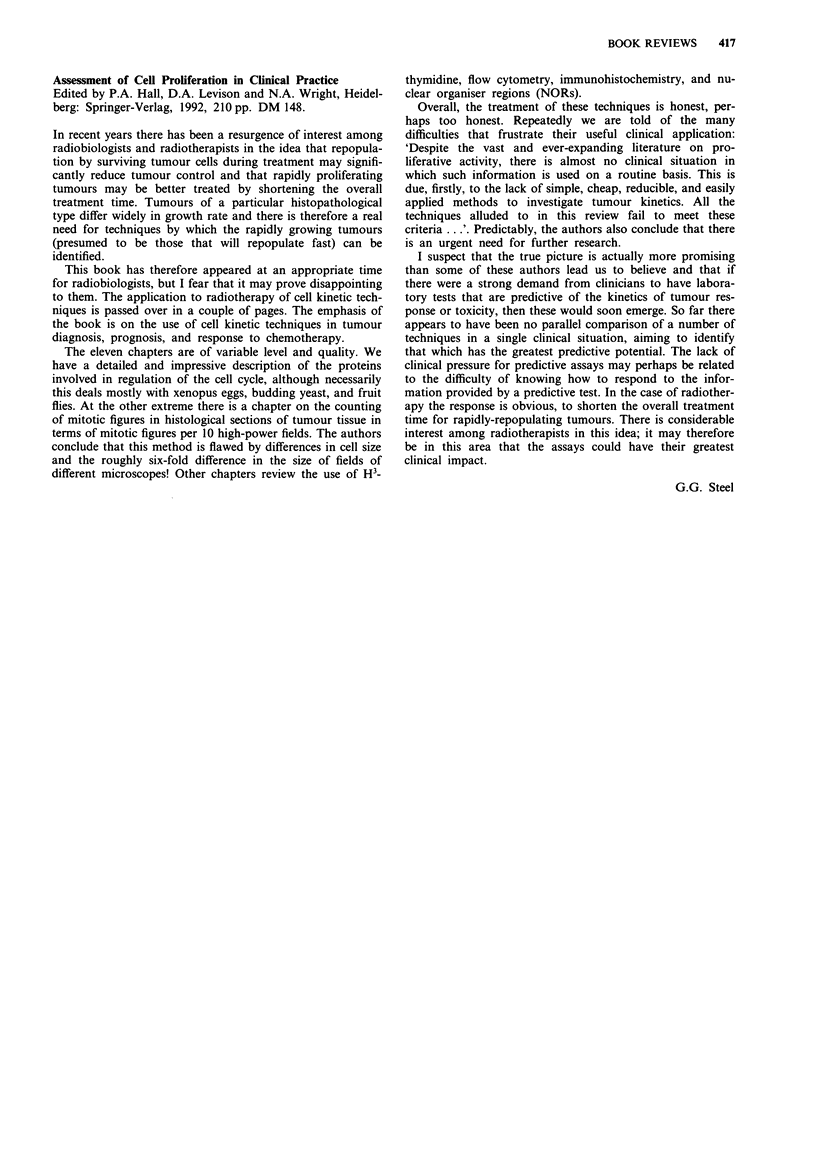# Assessment of Cell Proliferation in Clinical Practice

**Published:** 1992-08

**Authors:** G.G. Steel


**Assessment of Cell Proliferation in Clinical Practice**
Edited by P.A. Hall, D.A. Levison and N.A. Wright, Heidelberg: Springer-Verlag, 1992, 210 pp. DM 148.

In recent years there has been a resurgence of interest among radiobiologists and radiotherapists in the idea that repopulation by surviving tumour cells during treatment may significantly reduce tumour control and that rapidly proliferating tumours may be better treated by shortening the overall treatment time. Tumours of a particular histopathological type differ widely in growth rate and there is therefore a real need for techniques by which the rapidly growing tumours (presumed to be those that will repopulate fast) can be identified.

This book has therefore appeared at an appropriate time for radiobiologists, but I fear that it may prove disappointing to them. The application to radiotherapy of cell kinetic techniques is passed over in a couple of pages. The emphasis of the book is on the use of cell kinetic techniques in tumour diagnosis, prognosis, and response to chemotherapy.

The eleven chapters are of variable level and quality. We have a detailed and impressive description of the proteins involved in regulation of the cell cycle, although necessarily this deals mostly with xenopus eggs, budding yeast, and fruit flies. At the other extreme there is a chapter on the counting of mitotic figures in histological sections of tumour tissue in terms of mitotic figures per 10 high-power fields. The authors conclude that this method is flawed by differences in cell size and the roughly six-fold difference in the size of fields of different microscopes! Other chapters review the use of $H^3$-thymidine, flow cytometry, immunohistochemistry, and nuclear organiser regions (NORs).

Overall, the treatment of these techniques is honest, perhaps too honest. Repeatedly we are told of the many difficulties that frustrate their useful clinical application: 'Despite the vast and ever-expanding literature on proliferative activity, there is almost no clinical situation in which such information is used on a routine basis. This is due, firstly, to the lack of simple, cheap, reducible, and easily applied methods to investigate tumour kinetics. All the techniques alluded to in this review fail to meet these criteria . . .'. Predictably, the authors also conclude that there is an urgent need for further research.

I suspect that the true picture is actually more promising than some of these authors lead us to believe and that if there were a strong demand from clinicians to have laboratory tests that are predictive of the kinetics of tumour response or toxicity, then these would soon emerge. So far there appears to have been no parallel comparison of a number of techniques in a single clinical situation, aiming to identify that which has the greatest predictive potential. The lack of clinical pressure for predictive assays may perhaps be related to the difficulty of knowing how to respond to the information provided by a predictive test. In the case of radiotherapy the response is obvious, to shorten the overall treatment time for rapidly-repopulating tumours. There is considerable interest among radiotherapists in this idea; it may therefore be in this area that the assays could have their greatest clinical impact.

G.G. Steel